# Expression of 5 S rRNA genes linked to 35 S rDNA in plants, their epigenetic modification and regulatory element divergence

**DOI:** 10.1186/1471-2229-12-95

**Published:** 2012-06-20

**Authors:** Sònia Garcia, Lucie Crhák Khaitová, Aleš Kovařík

**Affiliations:** 1 Laboratori de Botànica, Facultat de Farmàcia, Universitat de Barcelona, Av. Joan XXIII s. n., Barcelona, Catalonia, 08028, Spain; 2 Institute of Biophysics, Academy of Sciences of the Czech Republic, Královopolská 135, Brno, CZ-6125, Czech Republic

**Keywords:** 5 S expression, *Artemisia*, Asteraceae, C-box, DNA methylation, Pol III promoter divergence, rDNA organization

## Abstract

**Background:**

In plants, the 5 S rRNA genes usually occur as separate tandems (S-type arrangement) or, less commonly, linked to 35 S rDNA units (L-type). The activity of linked genes remains unknown so far. We studied the homogeneity and expression of 5 S genes in several species from family Asteraceae known to contain linked 35 S-5 S units. Additionally, their methylation status was determined using bisulfite sequencing. Fluorescence in situ hybridization was applied to reveal the sub-nuclear positions of rDNA arrays.

**Results:**

We found that homogenization of L-type units went to completion in most (4/6) but not all species. Two species contained major L-type and minor S-type units (termed L_s_-type). The linked genes dominate 5 S rDNA expression while the separate tandems do not seem to be expressed. Members of tribe Anthemideae evolved functional variants of the polymerase III promoter in which a residing C-box element differs from the canonical angiosperm motif by as much as 30%. On this basis, a more relaxed consensus sequence of a plant C-box: (5’-RGSWTGGGTG-3’) is proposed. The 5 S paralogs display heavy DNA methylation similarly as to their unlinked counterparts. FISH revealed the close association of 35 S-5 S arrays with nucleolar periphery indicating that transcription of 5 S genes may occur in this territory.

**Conclusions:**

We show that the unusual linked arrangement of 5 S genes, occurring in several plant species, is fully compatible with their expression and functionality. This extraordinary 5 S gene dynamics is manifested at different levels, such as variation in intrachromosomal positions, unit structure, epigenetic modification and considerable divergence of regulatory motifs.

## Background

Nuclear ribosomal DNA (rDNA) encoding 5 S, 5.8 S, 18 S and 26 S rRNA belong to the most important housekeeping genes playing a central role in cell metabolism [1]. In plant genomes there may be from several hundred up to tens of thousands of highly homogeneous copies of each gene. A high copy number of these genes is probably important to ensure increased demand for proteosynthesis during plant development [2] but other functions, such as stabilization of the cell nucleus, have also been proposed [3]. Each large 35 S (45 S in animals) rDNA unit contains 18 S, 5.8 S and 26 S rRNA genes, the internal transcribed spacers (ITSs), and an intergenic spacer (IGS) (for review see [4]). The 35 S units are organized in tandem arrays at one or several loci. The 5 S rDNA encoding a 120-bp-long transcript has been traditionally considered to occupy separate chromosomal locations (hereafter S-type) in seed plants [5-9]. However, physical linkage of 5 S and 35 S genes predominates the organization of rDNA in streptophyte algae and early diverging land plants such as mosses [10,11]. These studies led to the hypothesis that “liberation” of 5 S genes from the 35 S unit might have occurred in a common angiosperm ancestor after the separation from early diverging plants. However, the linked arrangement of 35 S-5 S units (hereafter L-type) was later found in several species from the genus *Artemisia* (from family Asteraceae, considered one of the most phylogenetically derived groups of angiosperms), first based on cytogenetic evidence [12,13] and subsequently confirmed through molecular studies [14]. Additional studies showed that as many as 25% of Asteraceae members could have the unusual L-type arrangement of rDNA [15,16] and the L-arrangement has recently been found in the living fossil gymnosperm *Gingko biloba*[17]. Whether the L- or S-type was the ancestral rDNA status in angiosperm species remains to be determined. In most L-type genomes, the 5 S insertion occurs in the IGS within 1 kb downstream from the 26 S gene, and the corresponding transcript is encoded exclusively on the opposite DNA strand than the 26 S rRNA [15,16].

Although, in the cell, there has to be a stoichiometric ratio of rRNA molecules, fundamental differences exist with respect to the transcriptional regulation of individual genes. The large polycistronic 35 S transcript produced by RNA polymerase I (Pol I) is endonucleolytically processed to produce mature 18 S, 5.8 S and 26 S rRNA molecules [4]. The Pol I promoter, located within the 26-18 S intergenic spacer, binds to a complex of transcription factors [18]. In contrast, transcription of 5 S genes is carried out by RNA polymerase III (Pol III) which requires an internal promoter within the gene in addition to the TFIIIA, TFIIIB and TFIIIC transcription factors. The tripartite structure of the Pol III internal promoter comprises an A-box, an IE (internal element) and a C-box, elements that are highly conserved in plants and animals [19]. Epigenetic tools are another layer of expression control involved in the regulation of both types of rRNA genes. Silencing of non-transcribed gene copies is mediated by complex epigenetic mechanisms relying on chromatin modifications including DNA methylation [20,21].

While there is an increasing number of eukaryotic genomes with the L-arrangement of 5 S genes [10,11,22] their expression patterns and epigenetic modifications have not yet been investigated. The remaining issue is which (if any) of the linked genes are expressed and functional. In this work, we addressed the following questions:

1) Do the L- and S-type loci occur simultaneously in a given genome? If, so which of them contribute to 5 S expression?

2) How much homogeneous are the 5 S rRNA pools? Are regulatory elements conserved between the S- and L-type genes?

3) What are the DNA methylation and chromatin condensation patterns of genes with linked and unlinked arrangements?

We analyzed expression by RT-PCR, cloning and sequencing approaches. Bisulfite sequencing and FISH were used to determine DNA methylation and chromatin condensation levels.

## Methods

### Plant materials

We selected representative species known to evolve predominant L- or S-type arrangement of 5 S rDNA, in order to cover all three subtribes in which unusual linked arrangement arose (Anthemideae, Gnaphalieae and Heliantheae alliance) and whose 35-5 S units (IGS) had been previously sequenced. Leaf or seed material for the species *Artemisia absinthium*, *A. tridentata*, *Elachanthemum intricatum, Helianthus annuus,Helichrysum bracteatum*, *Gnaphalium luteoalbum*, *Matricaria matricarioides*, *Tagetes patula* (all Asteraceae) *and Linum alpinum* (Linaceae) were obtained either from wild populations or purchased. Plants were grown at the greenhouse of the Institute of Biophysics (Brno, CZ). Table 1 lists the provenance of the studied materials.

**Table 1:** List of species studied with an indication of their origin and collection data

**Species**	**Origin**
*Artemisia absinthium*	Spain: Catalonia, Setcases. S. Garcia and C. Ibarria ix. 2008.
*Artemisia tridentata* subsp. *vaseyana*	USA: Salt Creek Canyon, Juab. Co., Utah, USA. E. D. McArthur. 2008.
*Elachanthemum intricatum*	Mongolia: Southern Gobi. Sh. Dariimaa, Sh. Tsooj, J. Vallès and E. Yatamsuren. 2004.
*Gnaphalium luteoalbum*	Spain: Catalonia, Barcelona. Botanical Garden of Barcelona. S. Garcia. i.2007.
*Helianthus annuus*	Czech Republic: Bohemia, Dobříš. Nohel Garden. S. Garcia i.2009.
*Helichrysum bracteatum*	Czech Republic: Bohemia, Dobříš. Nohel Garden. S. Garcia i.2009.
*Matricaria matricarioides*	USA: Wyoming, Albany County. S. Garcia, E. D. McArthur, S. C. Sanderson and J. Vallès. ix.2008.
*Tagetes patula*	Czech Republic: Moravia, Brno. Near the Institute of Biophysics, T. Garnatje, x.2007.
*Linum alpinum*	Italy: Courmayeur. Giardino Botanico Alpino Saussurea (Ref. 335 W). Index Seminum 2009.

### Nucleic acids extractions, PCR and RT-PCR analysis

Genomic DNA (gDNA) was isolated following a CTAB protocol [14]. RNAs were extracted from leaf material using the RNeasy Plant Mini kit (Qiagen, Germany). The purified RNAs were treated with Turbo^TM^ DNase (Ambion, Applied Biosystems, USA) to get rid of traces of DNA contamination. To prepare cDNA, about 2 μg of RNA was reverse transcribed by Superscript reverse transcriptase (Invitrogen, USA) employing random nonamer primers. The gDNA and cDNAs were analyzed by PCR using the following primers: 5SgF: 5’-GGTGCGATCATACCAGCACT-3’, 5SgR: GGTGCAACACGAGGACTT-3’, IGS1_692_: 5’-CGGAACYACCAAAGCGAGTAAG-3’ (newly designed) and 26Spr1: AGACGACTTTAAATACGCGAC [23]. The 5 S regions delimited by the primer sets were amplified using Taq polymerase (Roche, Germany) with the following PCR program: one cycle at 94°C for 3 min; 29–35 (depending on amplicon) cycles at 55°C for 20 s, 72°C for 30 s, 92°C for 20 s; extension 72°C for 7 min. The PCR products were separated on a 1.2% agarose gel, stained with ethidium bromide and photodocumented (Ultralum, USA). Fragments corresponding to 5 S transcripts were cloned into the pDrive vector (Qiagen, Germany) and inserts were sequenced from both directions using the T7 and SP6 primers.

### Bisulfite sequencing

Bisulfite treatments were carried out on purified gDNA (~100 ng) using the EpiTect Bisulfite Kit (Qiagen, Germany). Primers amplifying the non-coding DNA strand designed with the aid of the BISPRIMER program [24] were as follows: forward primer 5'-GTTCGGATTCAAAAAAAGGGGT-3' and reverse primer 5'-CGATCATACCARCACTAAT-3' . The PCR program consisted on: one cycle at 94°C – 3 min; 35 cycles at 94°C – 20 s, 55°C – 20 s,72°C – 20 s; extension 72°C – 7 min. The PCR products were separated on 1.2% agarose gels, purified using a PCR purification kit (Macherey-Nagel, Germany) and cloned into a TA vector (pDrive, Qiagen, Germany). Positive clones were PCR-screened using vector SP6 and T7 primers. From 11 to 13 clones from each sample were sequenced (Eurofins MWG Operon, Germany).

### Fluorescence in situ hybridization (FISH)

The fresh root tips of *Helichrysum bracteatum* were pretreated with an aqueous solution of colchicine 0.05% at room temperature, for 2.5 - 4 h and fixed in 3:1 (v/v) ethanol: acetic acid. Protoplasts were obtained using cellulolytic enzymes (0.4% pectinase (Macerozyme R10, Duchefa, Holland), 0.4% cytohelicase [Sigma C8274, USA], and 0.4% cellulase [Onozuka RS, Duchefa, Holland) in citrate buffer), dropped onto microscope slides, frozen and desiccated using liquid nitrogen and 70% ethanol. Before FISH, the slides were pre-treated with 50 μg mL^-1^ RNaseA for 1 h at 37°C in a humid chamber. After washing three times in 2× SSC (2× standard saline citrate + 0.1% (w/v) sodium dodecyl sulfate), slides were dehydrated in an ethanol series (50%, 70% and 100%) and air-dried. Remnants of cytoplasm were removed with pepsin treatment (10 μg mL^-1^ in 10 mM HCl, 4 min room temperature). The slides were then washed, dehydrated in ethanol and fixed for 10 min in 3.7% formaldehyde in 1× PBS, washed three times in 2× SSC, dehydrated again and air dried. The hybridization mix contained 50 ng μL^-1^ (1000 ng/slide), of Cy3-labeled (GE Healthcare, Chalfont, St Giles, England) 5 S probe a 116 bp-long insert of the cloned tobacco 5 S rRNA gene [24], and 20 ng μL^-1^ (400 ng/slide) of 35 S rDNA probe (a 2.5 kb fragment of 26 S rRNA gene from tomato labeled with Spectrum Green, Abbott Molecular, IL, USA). The FISH hybridization mixture (20 μL per slide) consisted of labeled DNA probes, 4 μL of a 50% solution of dextran sulfate, 10 μL pure formamide, 0.5 μL TE buffer and 2 μL 20 × SSC, and it was denatured at 75°C for 15 min and immediately cooled on ice. This was applied to the slides, which were denatured in a thermocycler using a flat plate: 5 min at 75°C, 2 min at 65°C, 2 min at 55°C, 2 min at 45°C, and transferred into a prewarmed humid chamber and put into an incubator. After overnight hybridization at 37°C, the slides were washed with 2× SSC, then 0.1× SSC (high stringency), at 42°C for 10 min each followed by washes with 2× SSC, 4× SSC + 0.1% Tween 20 at room temperature. Slides were rinsed in PBS and mounted in Vectashield (Vector Laboratories, Burlinghame, CA, USA) containing DAPI (1 μg/mL^-1^). FISH signals were observed using an Olympus AX 70 fluorescent microscope equipped with a digital camera. Images were analyzed and processed using ISIS software (MetaSystems, Altlussheim, Germany).

### Bioinformatic methods

Sequences were assembled by BioEDIT Sequence Alignment Editor 7.0.9.0 [25] and aligned. The bisulfite data were processed and methylation density calculated using CyMATE software [26]. Secondary structure modeling was carried out through an online tool at the Mfold Web Server (The RNA Institute, College of Arts and Sciences, University of Albany, State University New York). Public database searches were carried out through BLAST [27]. Additional 5 S sequences for comparative purposes were downloaded from the 5 S RNA database [28].

## Results

### PCR on genomic DNA

**Figure 1:**
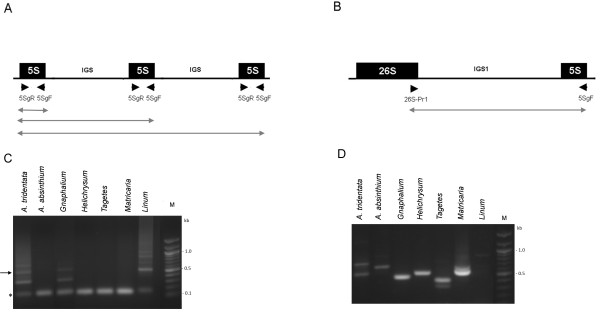
**PCR analysis of 5 S organization.** Schemes of separate **(A)** and linked **(B)** configuration of 5 S rDNA. Positions of primers are marked with arrowheads. The lines below indicate putative PCR products. **(C)** Analysis of separate organization using the 5SgF/5SgR primer pair. The arrow indicates position of the band extracted for subsequent cloning. Asterisk – position of a 5 S monomer (120 bp). **(D)** Analysis of linked organization using the 5SgF/26SPr1 primer pair. PCR products were separated in 1.2% agarose gels and detected by ethidium bromide fluorescence.

Previous Southern blot hybridization revealed large amounts of linked 35-5 S units in *Artemisia absinthium*, *Artemisia tridentata*, *Helichrysum bracteatum*, *Matricaria matricarioides* and *Tagetes patula*. Here, we wished to determine whether any unlinked (S-type) units were present in these genomes. For sensitivity we applied several PCR strategies (Figure 1) using primers specific for the 5 S and 26 S coding regions. In the case of a linked arrangement (5 S copies flanked by non-5 S DNA) only products corresponding to ~120-bp monomers would be amplified (Figure 1A). Correspondingly, the monomeric bands were amplified in *A. absinthium, H. bracteatum, M. matricarioides* and *T. patula*. In the case of tandem arrangement, mono and oligomeric products would be formed, the latter originating from polymerase read-through into neighboring units. This situation occurred in *Linum alpinum*, a species that typically evolved a separate arrangement of DNA units and which showed several oligomeric bands extending to a smear of an unresolved high-molecular-weight fraction (Figure 1C). Significantly, similar ladders though with a shorter periodicity were visualized in *A. tridentata* and *G. luteolbum* (weak). Except for *Linum,* reactions using 26SPr1-5SgF primers (Figure 1B) produced 1–2 bands of <1 kb confirming linked rDNA genotypes in all Asteraceae species studied (Figure 1D).

**Figure 2:**
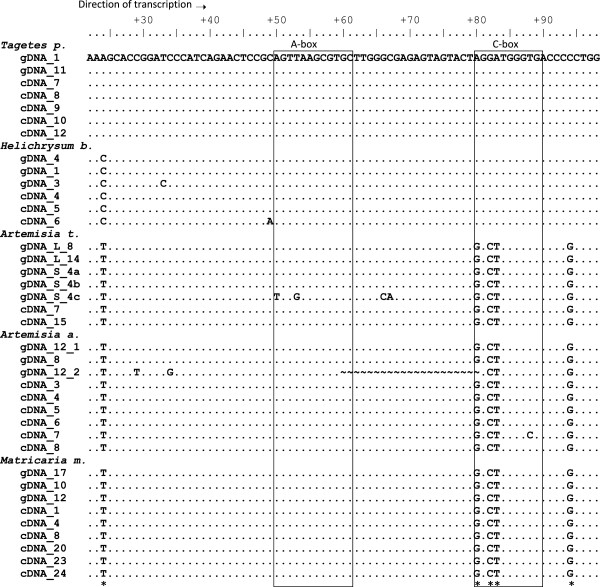
**Alignment of genomic and cDNA sequences.** The gDNAs were taken from the sequenced 26 S-18 S spacers and correspond mostly to 5 S rDNA1 present downstream of the 26 S gene [13]. One gDNA clone (12_2) was derived from a 5 S rDNA2 variant harboring an internal deletion. Three sequences (S_4a-c) originated from a cloned 5 S-5 S trimer from *A. tridentata*. Note, four mutations in the S4_c monomer. The cDNA clones were obtained from amplifications using the 5SgF and 5SgR primers. Conserved regulatory elements are boxed.

Next, we analyzed tandemly arranged genes in *A. tridentata* by cloning an oligomeric PCR product corresponding to a trimer (Figure 1C, arrow). Sequencing of three plasmid clones (Genbank: JX101914-JX101916) revealed that clones contained trimer (#4) and dimers (#3 and #7) of the 5 S gene. The characteristic feature of minor S-type units is an unusually short intergenic spacer (Additional file 1) whose size (58 bp) markedly differs from the average (100–900 bp) of 5 S-5 S spacers in plants [29,30]. The clones were highly homologous to each other and to the L-type copies (Figure 2). Two gene copies (clones 3 and 4) harbored mutations within the A-box regulatory element.

Thus, *A. tridentata* and possibly *G. luteoalbum* contain rDNA in both linked and separate configurations of 5 S genes. The ratio of gene copies is, however, shifted to linked 35 S-5 S units. Therefore this type of genomic arrangement was called as “L_s_”.

### Size and cloning analysis of 5 S cDNA sequences

**Figure 3:**
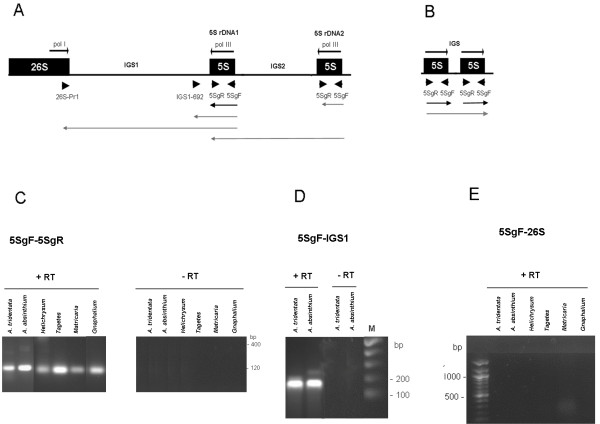
**RT-PCR analysis of 5 S expression. (A) Schematic representation of a part of the 35 S-5 S unit.** The 5 S rDNA1 is present in all species; additional 5 S rDNA2 is found in some *A. absinthium* units [14]. (**B**) Schematic representation of minor 5 S-5 S tandems in *A. tridentata*. Positions of primers are indicated by arrowheads. The arrowed lines below indicate putative transcripts primed from the Pol III promoters analyzed in (**C-E**). Black – coding region transcripts. Gray – long transcripts containing variable portions of IGS sequences. Cycling conditions: (**C**) – 29 cycles; (**D-E**) – 35 cycles. “+RT” and “-RT” indicate reactions with or without a reverse transcription step.

To study the expression of 5 S genes we analyzed RNA from five species with predominantly linked genotypes (*A. absinthiumA. tridentataH. bracteatumM. matricarioides* and *T. patula*) as shown in [15]. Several potential 5 S locus transcripts were examined by RT-PCR using different primer sets (Figure 3A, B). The 5SgF/5SgR primers would amplify a ~120 bp genic region corresponding to nearly an entire mature 5 S transcript. A longer product would correspond to polymerase read-through into the neighboring unit. These may originate either from independent tandem arrays (Figure 3B) or from the second incomplete 5 S rDNA2 copy in the 26 S-18 S spacer (Figure 3A). The second ~180 bp amplicon delimited by the IGS1_692_/5SgF primers involves the entire 5 S genic region plus about 60 bp of downstream IGS1 sequences. Finally, the third type of RT-PCR (26S_Pr1_/5SgF primer set) maps potential 5 S-IGS1-26 S transcripts. The genic 5SgF/5SgR primers actually amplified a ~120-bp fragment from all cDNA templates (Figure 3C) consistent with the typical length of a mature 5 S transcript. In contrast to genomic PCR, no oligomeric or high molecular weight fragments were visualized after the RT-PCR reaction. The IGS1_692_/5SgF primer set also amplified bands of expected size from *A. tridentata* and *A. absinthium* cDNA (Figure 3D). The 26 S _Pr1_/5SgF primers did not amplify the products of any of cDNAs (Figure 3E) while they did amplify a specific fragment from genomic DNA (Figure 1D).

The products of cDNA amplification were purified, cloned and sequenced. The alignment of cDNA and gDNA clones is shown in Figure 2. It is evident that, in each species, the cDNA clones were nearly identical to the gDNA clones derived from 5 S rDNA1. Minor differences were attributed to only a few random mutations. Similarly, alignment of longer spacer sequences (IGS1_692_/5SgF) of cDNA and genomic clones also revealed nearly complete identity (Additional file 2). Consequently, phylogeny dendrograms (ML, NJ) constructed from both genomic and cDNA sequences revealed species-specific clustering (not shown). While comparison of gDNA and cDNA clones failed to reveal substantial intragenomic polymorphisms, up to seven conserved variable sites (occurring in all units) were detected across the species. Surprisingly, three of them located to the Pol III promoter element (C-box) in position 80–89 (Figure 2).

### 5 S regulatory motifs and secondary structure

**Figure 4:**
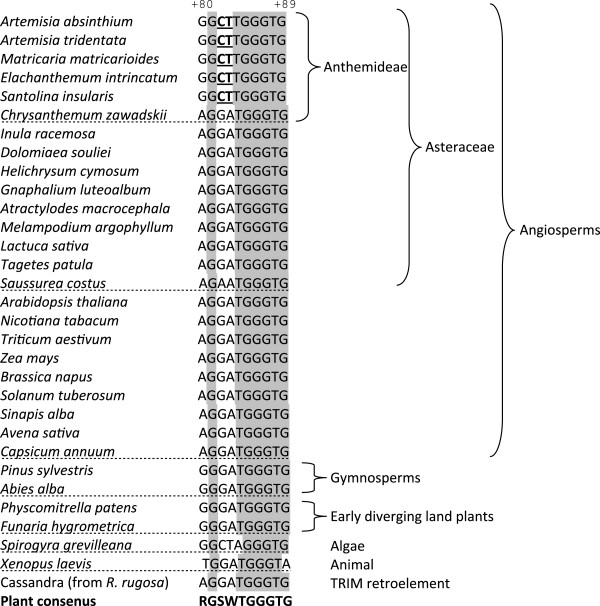
**Aligned C-box sequences from different plant and animal species.** Data were taken from the 5 S rDNA database [28] or the Genbank: EU816952, GU339175, JF277447, EU257402. Shaded letters indicate conserved nucleotides in plant genera. *Cassandra* is a non autonomous retroelement homologous to 5 S gene [45]. Variable nucleotides within the unique C*box are in bold underlined. Numbering is according to the *Arabidopsis* gene. Sequenced Asteraceae genes were reported in [46-48].

As mentioned earlier, the internal Pol III promoter comprises a tripartite motif composed of an A-box, internal regulatory element (IE) and the C-box. Sequencing of multiple clones in the different analyzed species revealed high level of conservation of A-box and IE elements (Figure 2). However, the third part of the internal regulatory region, the C-box, located at 80–89, was only partially conserved. There were three substitutions in the 5’ region: one A > G transition at a position +80 and two G > C and A > T transversions at +82 and +83, respectively. The 3’end of the C-box was invariant. All cDNA clones (excepting random non-fixed mutations) from *A. absinthiumA. tridentata* and *M. matricarioides* contained the same (5’-GGCTTGGGTG-3’) variant of the C-box (termed C*-box) whereas the other studied species displayed the canonical 5’-AGGATGGGTG-3’motif (Figures 2 and 4) [31]. The upstream sequences were less conserved but the TATA box at about −20 was present in all genomic clones [15] including the one originating from minor separate loci in L_s_ species. In addition, there were multiple dT terminators in each IGS1, one or two immediately downstream of the last 5 S gene nucleotide (Additional file 2) while only a single terminator was found in the S-type genomic clones from *A. tridentata* (Additional file 1). We did not identify any repeated elements (using the REPFIND tool, Vienna server, http://molbioltools.ca/Repeats_secondary_structure/server) within the IGS1, proposed to function in the termination of transcription with Pol I [32].

**Figure 5:**
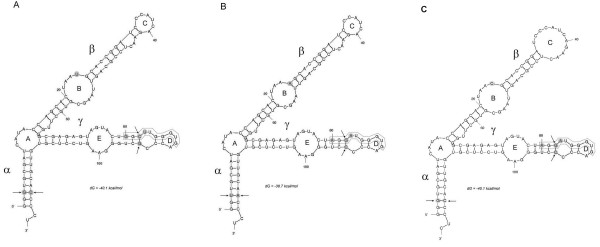
**Secondary structure models for 5 S rRNA from*A. absinthium*(A),*T. patula*(B) and*H. bracteatum*(C).** Variable nucleotides are highlighted by gray shading. Arrows indicate compensatory substitutions. The C-box element is boxed. Structural domains and loops are respectively in greek and latin letters following the nomenclature of [35,36].

The secondary structure of 5 S rRNA is believed to be important for its function on ribosomes, since its pseudogenes usually deviate from the typical Y-shaped molecule [33]. We wished to determine the influences of conserved substitutions (occurring in all clones of a given species) on the folding of RNA molecules. Using the web-based computer program Mfold Web Server [34], the 5 S rRNA secondary structures of three species (*A. absinthiumT. patula* and *H. bracteatum*) found to differ by several mutations were modeled (Figure 5). The alpha domain, considered to be the least conserved among land plants [11,28] showed a single polymorphic site at position +3. At this site, the G > T substitution was compensated by a C > A substitution at position +118, thus maintaining a stable number of hydrogen bonds. Position +24 within loop B (the beta domain) was the most variable, occupied either by A, C or T nucleotides. Polymorphism at this site seem to influence the size of loop B; the smallest being that of *Tagetes*. The gamma domain was formed by loop E constituting part of the highly conserved A-box, and a small terminal loop D containing part of the C-box. It is evident that species-specific mutations did not seem to markedly influence domain structure.

### DNA methylation analysis

**Figure 6:**
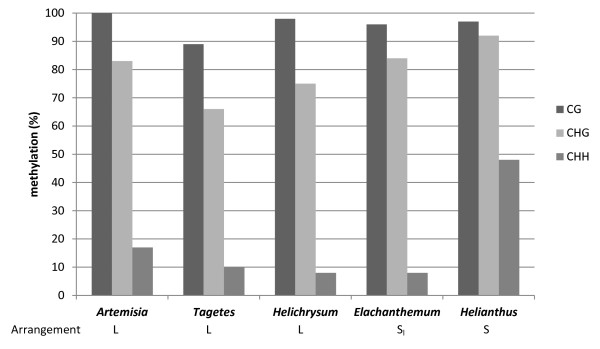
**Summary of bisulfite methylation analysis. The sequenced region involved the central part of the 5 S coding region.** The data were assembled from 11–13 clones per species (Additional file 4). Y-axis – average mC frequency along the clones expressed as a proportion of total Cs.

Using bisulfite sequencing we examined DNA methylation of 5 S genes occurring in two different genomic organizations with the aim to address the question whether the differential arrangement influences epigenetic patterns. We selected two representatives of both species with predominant linked (*Artemisia absinthium, Helichrysum bracteatum* and *Tagetes patula*) and separate (*Elachanthemum intricatum and Helianthus annuus*) rDNA arrangement and analyzed the 89 bp of the 5 S coding region that encompassed our primers (Figure 1A). After the bisulfite treatment the amplified PCR products were cloned and sequenced. The results of bisulfite analysis are presented as diagrams at a single clone resolution (Additional file 3) and summarized in Table 2 and Figure 6. The CG and CHG sites were more frequently methylated than the non-symmetrical CHH sites, which is typical for plant DNA [37]. There was also considerable variation between clones originating from the same individual (Table 2). For example, in *Tagetes*, a single clone (# 17) contained only two methylated Cs (11%) while there were clones with as much as 44% methylation. Substantial variation in methylation densities also occurred between the species.

**Table 2:** Relationship between genomic organization and DNA methylation of 5 S genes in the studied species

**Species**	^**1**^**Organization**	**Number of 5 S loci**	^**1**^**Position on chromosomes**	^**2**^**mC average**	**mC range**
*Artemisia absinthium*	L_S_	2	(sub-)terminal	46%	34-62%
*Tagetes patula*	L	7	(sub-)terminal	33%	11-44%
*Elachanthemum intricatum*	S_L_	n.d.	n. d.	39%	31-45%
*Helichrysum bracteatum*	L	1	(sub-)terminal	34%	16-43%
*Helianthus annuus*	S	5	pericentromeric	63%	30-96%
*Arabidopsis thaliana*	S	3	pericentromeric	^3^79%	^3^36-95%

^1^ The genomic organization and chromosomal position of 5 S rDNA is taken from [12-15,38] and from the Plant rDNA database [39].

^2^This work.

^3^ Methylation levels of coding region were determined by bisulfite sequencing. Data for *Arabidopsis* genes are from [40]; slightly lower values were reported in [41].

### FISH

**Figure 7:**
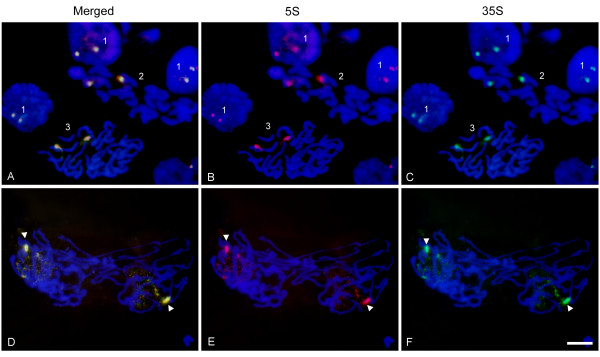
**Fluorescence*in situ*hybridization of 5 S (red) and 35 S rDNA (green) probes to*Helichrysum bracteatum*nuclei.** Pictures in the first row **(A, B & C)** show rDNA signals in interphase: (field 1), metaphase chromosomes (field 2), and a prophasic cell (field 3). The second row **(D, E & F)** shows late anaphase/early telophase. One rDNA homolog is highly condensed (arrowheads) whereas the other is spread throughout the nucleus and decondensed. Bar – 10 μm.

We analyzed the chromatin condensation patterns of 5 S and 35 S genes during different periods of the cell cycle (Figure 7). The 35 S and 5 S probes labeled with Spectrum green and cyanine Cy3, respectively, were hybridized to *Helichrysum bracteatum* interphase and metaphase nuclei. The signals of both probes colocalized to one pair of homologs (field 2, pictures A-C) indicating that there was a single 35 S-5 S locus in this species. Similarly, the prophase nuclei (field 3, pictures A-C) showed two colocalized signals on already condensed chromosomes. In interphase (field 1, pictures A-C) two dark bodies representing nucleoli were visible in most cells. The 35 S and 5 S signals tend to associate around the nucleolus and in some cases (upper field 1, pictures A-C) the decondensed signals spread into the nucleolus. Anaphase/telophase (D-F) chromosomes split into two chromatids. In each newly forming nucleus, one homolog started to decondense earlier than the other.

## Discussion

In higher eukaryotes (plants and animals), 5 S genes occur either as separate arrays (S-type) or, less frequently, linked to large 35 S units (L-type). While the expression of S-type genes has been thoroughly studied in the past, expression of L-type genes has not yet been addressed in any of these organisms. Here we show that in several representative plant species the linked 5 S genes are expressed and dominantly contribute to cellular 5 S rRNA pools.

### Low abundance of S-type loci in L-type genomes

Previous Southern blot and FISH analysis revealed largely homogenized 35 S-5 S units in several genera of the Asteraceae family [14][15]. The current PCR analysis revealed minor 5 S-5 S tandems along with dominant 35-5 S units in two species (*Artemisia tridentata* and *Gnaphalium luteoalbum*). Separate tandems likely originate from loci that did not hybridize with the 26 S probe on Southern blots [14]. Quantitative estimates suggest that they represent less than 10% of 5 S rDNA in L_s_ genomes [14]. Contrast to linked 35 S-5 S genes, the 5 S-5 S tandems contained mutations in the A-box element (2/7 monomers) suggesting the occurrence of non-functional copies. However, more clones need to be analyzed to obtain statistical support for differential mutation frequencies between the arrays. The absence of S-type tandems in other L-type genomes (Figure 1) further indicates their frequent loss and/or rapid replacement by linked units. The question arises as to the location of minor S-type loci on chromosomes. While FISH on metaphase chromosomes of *A. tridentata* failed to reveal separate 35 S and 5 S signals, in the interphase some sites were labeled more strongly with the 5 S or 35 S rDNA probe [14]. We therefore favor the hypothesis that minor 5 S-5 S tandems occur close to 35 S-5 S arrays or are interspersed between them.

Thus, four types of rDNA arrangement could be distinguished among different plant genera: (i) L-type, in which 35 S-5 S units are homogenized to completion (e.g., *Helichrysum, Matricaria and Tagetes*), (ii) L_S_-type, in which mostly linked 35 S-5 S units occur along with minor separate 5 S tandems (*Artemisia* and *Gnaphalium*), (iii) S_L_-type which is characterized by dominant independent 5 S-5 S tandems with a low abundance of 35 S-5 S units. *Elachanthemum intricatum* seems to be a representative of this group [15] and (iv) S-type, in which genomes contain independent 5 S tandems typical for most angiosperms.

### Transcription of 35 S-5 S arrays

The L_s_ genomes harbor dominant L-type units and minor S-type units. Nevertheless, both loci encode potentially transcribed genes. Since only a fraction of rRNA genes is usually transcribed in the cell (the rest is epigenetically inactivated) it was of interest to determine the origin of 5 S rRNA transcripts, particularly in the L_s_- and L-type genomes. The dominant expression of linked 5 S genes is supported by the following observations: (i) primary 5 S transcripts extending into IGS1 beyond the first termination signals were identified, which suggests that transcribed 5 S sequences actually stem from linked 5 S genes since a fraction of the IGS1 sequence is detected, (ii) no read-through transcripts were detected from tandemly arranged 5 S-5 S units in the L_s_-type species, *A. tridentata*, (iii) the RNAs derived from linked genes adopted a secondary structure typical of a functional molecule according to the RNA folding simulations preformed and finally, (iv) linked arrays contained undermethylated and decondensed chromatin fractions (Table 2 and Figure 6), likely corresponding to active genes. We therefore presume that the contribution from low abundant tandem arrays or dispersed 5 S genes in L_s_ and L-type genomes to total rRNA pools is minor, if any.

One consequence of 5 S and 35 S linkage could be the putative transcription of both genes arising from read-through with both RNA Pol I and Pol III enzymes (Figure 3). In mung bean (*Vigna radiata*), termination of 35 S transcription occurs within 65 bp and 315 bp downstream of the 3'end of the 26 S rRNA coding region [32]. In cucumber (*Cucumis sativus*), several termination signals in the IGS were observed, the first being 350 bp downstream of the 26 S gene [42]. In *Tagetes*, the functional 5 S rDNA1 insertion occurs just within ~200 bp downstream from the last 26 S gene nucleotide (Additional file 2 and [13]), providing the possibility of formation of a long 35 S-5 S precursor and perhaps a double stranded RNA. However, we were unable to identify any transcripts containing both 26 S and 5 S sequences (Figure 3). Thus, the transcription of both genes is probably efficiently terminated in the IGS1 and/or genes are compartmentalized in cell nucleus (discussed further below).

As previously noticed [14], some genes in *A. absinthium* contain a second 5 S insertion (5SrDNA2) located distally to the 26 S gene. However, the PCR product corresponding to the 5 S rDNA2 transcription (Figure 2) was not detected among the sequenced clones, supporting the hypothesis that it may represent a pseudogene and therefore is not transcribed. Nevertheless, the duplication may have evolutionary significance since a similar duplication with one functional and one non-functional 5 S copy was observed in horsetail, *Equisetum hyemale*[11]. Apparent parallelism may point to a common mechanism of 5 S integration, and/or similar selection pressures in different organisms maintaining gene functionality.

### Evolution of pol III promoter variants: An updated plant C-box consensus sequence

Unlike most other genes, 5 S rDNA contains essential regulatory elements within the internal controlling region (ICR). As a consequence, the 5 S rRNA carries the promoter sequences of the genes from which it is transcribed allowing the study of regulatory elements among cDNA sequences. The sequence and position of these elements (A- and C-box, Figure 2) is highly conserved across eukaryotes [31]. It was therefore surprising that several closely related species from tribe Anthemideae evolved a variant of the C-box (C*) that differed from the angiosperm consensus by as much as 30% (Figure 4). The motif was present in cDNA clones of respective species, and full congruence between cDNA and gDNA sequences was found, with little or no variation within the genome. Thus, units carrying the C*-box variant appeared to be functional. The closest relative of the C*-box was found in the algae *Spirogyra*. We therefore propose a revised, more relaxed version of the plant C-box consensus motif, written onwards as 5’-RGSWTGGGTG-3’. Most variation is located at the 5’ half of the box. It is surprising that mutations in this region actually lead to reduced transcription in *Arabidopsis*[43]. Specifically, the +82 G > T and +84 T > C substitutions caused, respectively, partial or complete loss of transcription; in our new version, the C*-box contained C at +82 while the T at +84 remained invariant. This suggests that +84 T might be critical for C-box functionality while the other three nucleotides in the 5’ half can be more variable. Besides, the secondary structure does not seem to be influenced by the C-box polymorphisms (Figure 5) suggesting that the rRNA-TFIIIA interactions may not be impaired. However, since two out of three mutations were non-compensatory, their influences on tertiary structure [44] and binding of other factors cannot be excluded.

There does not seem to be a simple correlation between the occurrence of the promoter variants and the genomic arrangement of rRNA genes. For example, the C*-box is found in some, but not all L-type species. The phylogeny study suggests its preferential occurrence in the tribe Anthemideae, but again, not all members seem to bear it (Figure 4). Sequence divergence may rather reflect the overall dynamics of locus undergoing frequent elimination/homogenization cycles in this group of plants. In this context, Asteraceae species show diverse positions of rDNA loci on chromosomes [13,16,49], substantial genome size variation [50], certain phylogenetic incongruence between 35 S and 5 S markers [51], and even rearrangements between telomeric and rDNA repeats [52]. The C*-box variant appears to be of recent origin that perhaps evolved after the divergence of Anthemideae from the rest of Asteraceae less than few million years ago [53]. To gain a better understanding of such C-box divergence, it will be interesting to analyze 5 S promoters in many other Asteraceae species, as well as the transcription factors binding to them, in order to detect their possible co-evolution. Of note, TFIIIA is known to evolve rapidly (yeast and animal genes share only 20% similarity) and splicing of its primary transcript seems to be influenced by an exonized 5 S insertion in plants [54].

### Similar DNA methylation patterns of linked and separate 5 S rDNA

Both S- and L-type species showed CG, CHG and CHH methylation patterns typical for plant repetitive DNA [37]. Consistently, the methylation density at different motifs had the identical tendency descending in this order: CG > CHG > CHH in line with previous studies of 5 S methylation [41,55]. One can conclude that a relatively high level of methylation (usually higher than genome average) is not linked to tandem arrangement but also occurs when 5 S genes are organized as single or low copy insertions. In other words, the tandem arrangement does not seem to be essential for 5 S methylation. Within the tandemly arranged units, genes with low or no methylation levels are considered active while highly methylated genes are heterochromatic and inactive [20]. Variation in methylation density between clones might reflect epiallelic heterogeneity of arrays. Significantly undermethylated genes with as little as 11% methylation (Table 2) were detected possibly originating from the highly active part of 35 S-5 S arrays. A relatively high level of methylation, particularly at non-CG motifs, was found in *Helianthus* (S-type). *Helianthus annuus* shows pericentromeric location of 5 S rDNA [15,38] while the less methylated 5 S genes in *Tagetes patula* and *Artemisia absinthium* are located at (sub-)telomeric positions [12,15]. In this sense, 5 S units located proximally to centromeres in *Arabidopsis* were more methylated than other distally located genes [56].

### Relationship between chromatin condensation and expression of rDNA units

It is known that RNA Pol I (which transcribes the 35 S genes) occurs in the nucleolus while RNA Pol III (which transcribes 5 S genes) is a nucleoplasmic protein. Thus, a single 35 S-5 S unit actively transcribed by one polymerase cannot be transcribed at the same time by the other polymerase. Strict compartmentalization of 35 S and 5 S transcription may also explain our failure to detect products of bidirectional 26 S-5 S transcription (Figure 3). Several models of spatial control of rDNA expression can be envisaged. First, there could be frequent reshuffling of genes between the nucleolus and nucleoplasm. This is unlikely considering that different transcription machineries are needed to execute the transcription of 5 S and 35 S genes. The second possibility is that a part of the megabase-sized array could be transcribed by polymerase I while another part is transcribed with Pol III. Certainly, the L-type genomes harbor enough genes (several thousand copies [15]) allowing the separation of arrays into transcription domains. Finally, regulation may occur at the level of individual chromosome sites. For example, one chromosome homolog could be involved in organizing the nucleolus and the transcription of 35 S genes while the other homolog transcribes 5 S genes. The FISH experiment in Figure 7 may provide some experimental support for this hypothesis. In late anaphase/telophase of *Helichrysum bracteatum* the nucleoli were apparently assembled on one highly decondensed homolog, while the other was highly condensed and probably not involved in nucleolus assembly. Such a dramatic difference in condensation patterns was not seen in interphase in which rDNAs on both homologs were condensed and associated with the nucleolar periphery (Figure 7A-C). Interestingly, TFIIIA factor essential for 5 S transcription seems to be concentrated at several nuclear foci including the nucleolus in *Arabidopsis*[57] suggesting that transcription of linked 5 S genes may occur in a close proximity of the nucleolus.

## Conclusions

With the present study, evidence was obtained for a dominant contribution of linked 5 S genes to the overall 5 S rRNA pools in species with completely or partially homogenized 35 S-5 S arrays, that is, 5 S genes are entirely transcribed from these linked arrays. The unusual sequence variation found in the internal regulatory elements of 5 S genes seems to be fully compatible with transcription, and considering these variations, an updated C-box consensus sequence has therefore been proposed. The methylation patterns of linked genes seem to be similar to their unlinked counterparts. As for the nuclear topology, the 35 S-5 S arrays closely associate with the nucleolus, suggesting that 5 S transcription may occur in close proximity to the nucleolus, possibly at its periphery.

## Abbreviations

gDNA, Genomic DNA; cDNA, Complementary DNA; IGS, Intergenic spacer between the 26 S and 18 S rRNA genes; L-type, Linked arrangement of the 35 S and 5 S RNA genes; S-type, Separate arrangement of the 35 S and 5 S RNA genes; Ls-type, Linked arrangement with minor contribution of S arrangement; Sl-type, Separate arrangement with minor contribution of L arrangement; Pol I, RNA polymerase I; Pol III, RNA polymerase III; ICR, Internal controlling region; FISH, Fluorescent in situ hybridization.

## Competing interests

The authors declare that they have no competing interests.

## Authors' contributions

SG and AK designed the study and wrote the paper. SG carried out most of the molecular biology and cytogenetic experiments; AK carried out the molecular and bioinformatic studies and drafted the paper, LCK isolated RNA and prepared cDNAs. All authors read and approved the final manuscript.

## Supplementary Material

Additional file 1 **Sequencing of 5 S oligomers from *A. tridentata.*** Alignment of sequenced clones. Coding regions are in bold letters. Boxes A and C are in yellow shading. The TATA box and termination signals are in red and blue, respectively. Asterisks indicate mutations. Click here for file

Additional file 2 **Alignment of long 5 S-IGS1 clones from A*. absinthium***. Alignment of 3 cDNA and 2 genomic (gDNA) clones containing 5 S genic and intergenic sequences. Termination signals are underlined. Click here for file

Additional file 3 **Structure of the 26 S-5 S intergenic spacer.** Alignment of genomic clones. The first ~30 nucleotides represent the 3’end of the 26 S gene. The last nucleotide belongs to the 5 S coding region. Strand reading the 35 S gene is shown; 5 S is encoded by the bottom strand. Termination signals for Pol III transcription are highlighted. Note spacer length heterogeneity. Click here for file

Additional file 4 **Bisulfite analysis of the 5 S rDNA genic region(central part).** Description: CyMATE program outputs from sequencing of non coding strands are shown. Filled symbols – methylated Cs; empty symbols non-methylated Cs. The numbers below the diagrams indicate C residues in the alignments. Gaps in matrices were caused by sequence polymorphisms. Click here for file
