# Supplementary material for: Expression of 5 S rRNA genes linked to 35 S rDNA in plants, their epigenetic modification and regulatory element divergence
Source: BMC Plant Biol. 2012 Jun 20;12:95. doi: 10.1186/1471-2229-12-95 (PMC3409069; doi:10.1186/1471-2229-12-95)
Supplement: Additional file 1 — Sequencing of 5 S oligomers from A. tridentata. Alignment of sequenced clones. Coding regions are in bold letters. Boxes A and C are in yellow shading. The TATA box and termination signals are in red and blue, respectively. Asterisks indicate mutations. [file 1471-2229-12-95-S1.pdf]

```
              ....|....| ....|....| ....|....| ....|....| ....|....| ....|....|
5S gene, copy 1    5         15         25         35         45         55
4T_SP         GGTGCGATCA TACCAGCACT AATGCACCGG ATCCCATCAG AACTCCGCAG TTAAGCGTGC
7T_SP         ---------- TACCATAACT AATGCACCGG ATCCCATCAG AACTCCGCAG TTAAGCGTGC
3T_SP         ---GCGATCA TACCAGCACT AATGCACCGG ATCCCATCAG AACTCCGCAG TTAAGCGTGC
                            **

              ....|....| ....|....| ....|....| ....|....| ....|....| ....|....|
                    65         75         85         95        105        115
4T_SP         TTGGGCGAGA GTAGTACTGG CTTGGGTGAC CCGCTGGGAA GTCCTCGTGT TGCACCCCTT
7T_SP         TTGGGCGAGA GTAGTACTGG CTTGGGTGAC CCGCTGGGAA GTCCTCGTGT TGCACCCCTT
3T_SP         TTGGGCGAGA GTAGTACTGG CTTGGGTGAC CCGCTGGGAA GTCCTCGTGT TGCACCCCTT

              ....|....| ....|....| ....|....| ....|....| ....|....| ....|....|
                   125        135        145        155        165        175   5S gene, copy 2
4T_SP         TTTTTGAATC CGAATGGAAT CCTACTATAT AAAAGGATAG ATTGCGAATA CTATGACGGG
7T_SP         TTTTTGAATC CAAATGGAAT CCTACTATAT AAAAGGATAG ATTGCGAATA CTATGACGGG
3T_SP         TTTTTTAATC CGAATGGAAT CCTACTATAT AAAAGGATAG ATTGCGAATA CTATGACGGG
                   *          *

              ....|....| ....|....| ....|....| ....|....| ....|....| ....|....|
                   185        195        205        215        225        235
4T_SP         TGCGATCATA CCAGCACTAA TGCACCGGAT CCCATCAGAA CTCCGCAGTT AAGCGTGCTT
7T_SP         TGCGATCATA CCATAACTAA TGCACCGGAT CCCATCAGAA CTCCGCAGTT AAGCGTGCTT
3T_SP         TGCGATCATA CCAGCACTAA TGCACCGGAT CCCATCAGAA CTCCGCAGTT AAGCATGCTT
                            **                                          *

              ....|....| ....|....| ....|....| ....|....| ....|....| ....|....|
                   245        255        265        275        285        295
4T_SP         GGGCGAGAGT AGTACTGGCT TGGGTGACCC GCTGGGAAGT CCTCGTGTTG CACCCCTTTT
7T_SP         GGGCGAGAGT AGTACTGGCT TGGGTGACCC GCTGGGAAGT CCTCGTGTTG CACC......
3T_SP         GGGCGAGAGT AGTACTGGCT TGGGTGACCC GCTGGGAAGT CCTCGTGTTG CACC......

              ....|....| ....|....| ....|....| ....|....| ....|....| ....|....|
                   305        315        325        335        345        355  5S gene, copy 3
4T_SP         TTTGAATCCG AATGGAATCC TACTATATAA AAGGATAGAT TGCGAATACT ATGACGGGTG
7T_SP         .......... .......... .......... .......... .......... ..........
3T_SP         .......... .......... .......... .......... .......... ..........

              ....|....| ....|....| ....|....| ....|....| ....|....| ....|....|
                   365        375        385        395        405        415
4T_SP         CGATCATACC AGCACTAATG CACCGGATCC CATCAGAACT CCGCTGTGAA GCGTGCTTGG
7T_SP         .......... .......... .......... .......... .......... ..........
3T_SP         .......... .......... .......... .......... .......... ..........
                                                                  *

              ....|....| ....|....| ....|....| ....|....| ....|....| ....|
                   425        435        445        455        465        475
4T_SP         CAGAGAGTAG TACTGGCTTG GGTGACCCGC TGGGAAGTCC TCGTGTTGCA CC
7T_SP         .......... .......... .......... .......... .......... ..
3T_SP         .......... .......... .......... .......... .......... ..
```
