# Supplementary material for: Expression of 5 S rRNA genes linked to 35 S rDNA in plants, their epigenetic modification and regulatory element divergence
Source: BMC Plant Biol. 2012 Jun 20;12:95. doi: 10.1186/1471-2229-12-95 (PMC3409069; doi:10.1186/1471-2229-12-95)
Supplement: Additional file 2 — Alignment of long 5 S-IGS1 clones from A. absinthium. Alignment of 3 cDNA and 2 genomic (gDNA) clones containing 5 S genic and intergenic sequences. Termination signals are underlined. [file 1471-2229-12-95-S2.pdf]

Direction of transcription

```
              +30        40        50        60        70        80        90        100       110
         ....|....|....|....|....|....|....|....|....|....|....|....|....|....|....|....|....|....|
gDNA_12  TAATGCACCGGATCCCATCAGAACTCCGCAGTTAAGCGTGCTTGGGCGAGAGTAGTACTGGCTTGGGTGACCCGCTGGGAAGTCCTCGTG
gDNA_8   ..........................................................................................
cDNA_7   ..........................................................................................
cDNA_8   ..........................................................................................
cDNA_10  ..........................................................................................
              120       130       140       150       160       170       180
         ....|....|....|....|....|....|....|....|....|....|....|....|....|....|
gDNA_12  TTGCACCCCTTTTTTTGAATCCGAACGTCACAACTTTTTTTTTCCGGCTTACTCGCTTTTGGTAGTTCCG
gDNA_8   ......................................................................
cDNA_7   ..................................--...........................G......
cDNA_8   ......................................................................
cDNA_10  ..................................-...................................
```

5S genic - end
