# Supplementary material for: Expression of 5 S rRNA genes linked to 35 S rDNA in plants, their epigenetic modification and regulatory element divergence
Source: BMC Plant Biol. 2012 Jun 20;12:95. doi: 10.1186/1471-2229-12-95 (PMC3409069; doi:10.1186/1471-2229-12-95)
Supplement: Additional file 3 — Structure of the 26 S-5 S intergenic spacer. Alignment of genomic clones. The first ~30 nucleotides represent the 3’end of the 26 S gene. The last nucleotide belongs to the 5 S coding region. Strand reading the 35 S gene is shown; 5 S is encoded by the bottom strand. Termination signals for Pol III transcription are highlighted. Note spacer length heterogeneity. [file 1471-2229-12-95-S3.pdf]

```
                     10        20        30        40        50        60        70        80
             ....|....|....|....|....|....|....|....|....|....|....|....|....|....|....|....|
A. absinth   TTCAGCCCTGCGTCGCTCAGATTCGTCCCTCCCCCCCAAAACATCCCACTCCATTTTCGTTTTTACACTTTGCCCAGAG
A. absinth   TTCAGCCCTGCGTCGCTCAGATTCGTCCCTCCCCCCCAAAACATCCCACTCCATTTTCGTTTTTACACTTTGCCCAGAG
A.tridenta   TTCAGCCATGCGTCGCTCAGATTCGTCCCTCCCCCCAAAAATACATCCCACTCCATTTTGGTTTCTTGCACTTTGCCCGG
Matricaria   TTCAGCCCTGCGTCGCTCAGATTCGTCCCTCCCCCCCCAAAACACATCTCACCTCATTTCCTTCCTTTGTTTTTTCCTAG
Matricaria   TTCAGCCCTGCGTCGCTCAGATTCGTCCCTCCCCCCCCAAAACACATCTCACCTCATTTCCTTCCTTTGTTTTTTCCTAG
Matricaria   TTCAGCCCTGCGTCGCTCAGATTCGTCCCTCCCCCCCCAAAACACATCTCACCTCATTTCCTTCCTTTGTTTTTTCCTAG
Helichryss   TTCAGCCCTATGTCGCTCAGATTCGTCCCTCCCCCTCAAAAACACATCTCTAAAATCATCTATATTGTTTTTAAAGAGGC
Helichryss   TTCAGCCCTATGTCGCTCAGATTCGTCCCTCCCCCTCAAAAACACATCTCTAAAATCATCTATATTGTTTTTAAAGAGGC
Gnaphalium   TTCAGCCCTATGTCGCTCAGACTCGTCCCTCCCCCTCAAAAAACACATCCCTAAATTCATCTATATTTCTTTTCAAGAGG
Gnaphalium   TTCAGCCCTATGTCGCTCAGATTCGTCCCTCCCCCTCAAAAAACACATCCCTAAATTCATCTATATTTCTTTTCAAGAGG
Tagetes_1    TTCAGCCCTTCGTCGCTCAGATTCGTCCCTCCCCCCTTACAGCCCCCCACACTCTTTGCCCCCATGGCAACTAACCTAT
Tagetes_11   TTCAGCCCTTCGTCGCTCAGATTCGTCCCTCCCCCCTTACAGCCCCCCACACTCTTTGCCCCCATGGCAACTAACCTAT
                       90       100       110       120       130       140       150       160
             ....|....|....|....|....|....|....|....|....|....|....|....|....|....|....|....|
A. absinth   GTTACCCCTCCTACATACTGAAGAATTTTACCAAGTGTTGAGAAATTTGTTCATAAATCGTCGGGCGAGGACTTTTACCA
A. absinth   GTTACCCCTCCTACATACTGAAGAATTTTACCAAGTGTTGAGAAATTTGTTCATAAATCGTCGGGCGAGGACTTTTACCA
A.tridenta   AGGTTTACCTACGTAATAACGGTAAGAATTTTACCAAGTGCTGAGAAATTTGTTCATAAATCGTCGAGCGATAACGTAGC
Matricaria   AGGCTATCTACATAGAAACTCAATAATTTGACTAAGTGTTGAGAAATTTGTTCATACATCGTCGGCGTGTTGGCGTTTCT
Matricaria   AGGCTATCTACATAGAAACTCAATAATTTGACTAAGTGTTGAGAAATTTGTTCATACATCGTCGGCGTGTTGGCGTTTCT
Matricaria   AGGCTATCTACATAGAAACTCAATAATTTGACTAAGTGTTGAGAAATTTGTTCATACATCGTCGGCGTGTTGGCGTTTCT
Helichryss   TGCGTGCCAATAATTGGTGAAAATATCACCAAGTGTTGGAGAATTTGACATGCGCCATCACTACGTGTTATGGCGCTCGG
Helichryss   TGCGTGCCAATAATTGGTGAAAATATCACCAAGTGTTGGAGAATTTGACATGCGCCATCACTACGTGTTATGGCGCTCGG
Gnaphalium   CTGCGCACCAATAATTTGTGATATTTCAAGTGTTGGAGAATTTGACATATCACACTACGTGTTATGACCGCGTCATCCCA
Gnaphalium   CTGCGCACCAATAATTTGTGATATTTCAAGTGTTGGAGAATTTGACATATCACACTACGTGTTATGACCGCGTCATCCCA
Tagetes_1    AAGGATATGTTTCAGAGGCTGGTTTCTCACACTTACGAAAATTCACTAAGTGTTGTACATAATGAGTTAGGATTTCTAAA
Tagetes_11   AAGGATATGTTTCAGAGGCTGGTTTCTCACACTTACGAAAATTCACTAAGTGTTGTACATAATGAGTTAGGATTTCTAAA
              170       180       190       200       210       220       230       240
             ....|....|....|....|....|....|....|....|....|....|....|....|....|....|....|....|
A. absinth   AGGGATGCTTGACGTGTTGGTCGTTTCGTGACGCGTGGACATCCATCATCGTTGGTCACACATCATTACAAGTGATGGTG
A. absinth   AGGGATGCTTGACGTGTTGGTCGTTTCGTGACGCGTGGACATCCATCATCGTTGGTCACACATCATTACAAGTGATGGTG
A.tridenta   AAGTGTTGGTGGTTTCGTGACGTGTGGCCATCCATCATCGCTGGTTAGACATCGTTACAAGTGATGATGGTGGTTGCTTG
Matricaria   TGTCTGCGCCTACGAAGAGGCTCTGCAAGCGTTGGTAAAATCTTTCCCAGAGTTTAAAGACCCAATAATTTGACTAAGTG
Matricaria   TGTCTGCGCCTACGAAGAGGCTCTGCAAGCGTTGGTAAAATCTTTCCCAGAGTTTAAAGACCCAATAATTTGACTAAGTG
Matricaria   TGTCTGCGCCTACGAAGAGGCTCTGCAAGCGTTGGTAAAATCTTTCCCAGAGTTTAAAGACCCAATAATTTGACTAAGTG
Helichryss   ATTCATCCACGTGTCGTCCCTCCCGCTCAAAAATCATCGATATTATTTTTCAAGAGGCTGCGTGTTATGCGATGCTCATG
Helichryss   ATTCATCCACGTGTCGTCCCTCCCGCTCAAAAATCATCGATGTTATTTTTCAAGAGGCTGCGTGTTATGCGATGCTCATG
Gnaphalium   CATCCCTACAAACATCTATATTATTTTTCACCAAGTGTTGGAGAATTTGCCCACGCGTCGCTCAGATTTGTCCCTCCCGC
Tagetes_1    GACACAGATAAAGTTTTGACCCCGACATCCGATCTGATGGTGGGACTGAAAAAAAAAAAAAAGATACACTAAAA 5S rDNA1
Tagetes_11   GACACAGATAAAGTTTTGACCCCGACATCCGATCTGATGGTGGGACTGAAAAAAAAAAAAAAAGATACACTAAAAA 5S rDNA1
```

```
                     250       260       270       280       290       300       310       320
             ....|....|....|....|....|....|....|....|....|....|....|....|....|....|....|....|
A. absinth   GTTGCTTGACGTGCGAAAATCCATTATTCTTCCTGACATTGTTCCTAAAGGTTAGCTACGCAAGAACTCGGAAAGACCAA
A. absinth   GTTGCTTGACGTGCGAAAATCCATTATTCTTCCTGACATTGTTCCTAAAGGTTAGCTACGCAAGAACTCGGAAAGACCAA
A.tridenta   ACGTGCGGCTATCCATTATTCTTCCCTACACTGTTGTTCCCTAAAGGTTAGCTACGCAAGAACTYGGAAAGACCCCGGAA
Matricaria   CAAGCGTTGTTCATACATCGTTGGCGTGTTGGCCGTTTCTTGACTACGCCTACGAAGAGTCGCACGTAAGAGCTCGGAAA
Matricaria   CAAGCGTTGTTCATACATCGTTGGCGTGTTGGCCGTTTCTTGACTACGCCTACGAAGAGTCGCACGTAAGAGCTCGGAAA
Matricaria   CAAGCGTTGTTCATACATCGTTGGCGTGTTGGCCGTTTCTTGACTACGCCTACGAAGAGTCGCACGTAAGAGCTCGGAAA
Helichryss   ACGCCTTCACCATGCTTAGGCGGTAGTGGGGAAGTTCGTACTCGGAAAAAAAAAAAAAA 5SrDNA1
Helichryss   ACGCCTTCACCATGCTTAGGCGGTAGTGGGGAAGTTCGTACTCGGAAAAAAAAAAAAAAAAA 5SrDNA1
Gnaphalium   TCAGAAACACATCCCAAAAATCATCGATATTATTTTTCATAGTCAGAGGCTGCGATGCTGATGACGCCTTCACCATGCTT
Gnaphalium   TCAGAAACACATCCCAAAAATCATCGATATTATTTTTCATAGTCAGAGGCTGCGATGCTGATGACGCCTTCACCATGCTT

                     330       340       350       360       370       380       390       400
             ....|....|....|....|....|....|....|....|....|....|....|....|....|....|....|....|
A. absinth   GTGTTGTCCAACATTTTTGAGAAACCGTTGGAGGGATGCTGGGTGGTTTATTACCACCATCCATTCTTCGTAGTCTCAAA
A. absinth   GTGTTGTCCAACATTTTTGAGAAACCGTTGGAGGGATGCTTGGTGGTTTATTACCACCATCCATTCTTCGTAGTCTCAAA
A.tridenta   CCACCAAAAAGCGAGTAAGAAGGGAAAAAAAAAGTTGTGATGTTCGGATCAAAAAAA 5SrDNA1
Matricaria   AACCGAGCAAGACACTATTTTAAAAAAAAATGTTGGATGATGTTAACGTGTCGGTGGTTTATGACTAAGAATATGAAAAA
Matricaria   AACCGAGCAAGACACTATTTTAAAAAAAAATGTTGGATGATGTTAACGTGTCGGTGGTTTATGACTAAGAATATGAAAAA
Matricaria   AACCGAGCAAGACACTATTTTAAAAAAAAATGTTGGATGATGTTAACGTGTCGGTGGTTTATGACTAAGAATATGAAAAA
Helichryss
Helichryss
Gnaphalium   AGGCGGTAGTGGGGTAGTACGTACTCAGGAGGGAAAAAAAAAAA 5SrDNA1
Gnaphalium   AGGCGGTAGTGGGGTAGTACGTACTCAGGAGGGAAAAAAAAAA 5SrDNA1

                     410       420       430       440       450       460       470
             ....|....|....|....|....|....|....|....|....|....|....|....|....|....|....|.
A. absinth   AGGATTGTGACGTCCTCGGAACTACCAAAAGCGAGTAAGCCGGAAAAAAAAAGTTGTGACGTTCGGATTCAAAAAA 5SrDNA1
A. absinth   AGGATTGTGACGTCCTCGGAACTACCAAAAGCGAGTAAGCCGGAAAAAAAAAGTTGTGACGTTCGGATTCAAAAAA 5SrDNA1
A.tridenta
Matricaria   GA 5SrDNA1
Matricaria   GA 5SrDNA1
Matricaria   GA 5SrDNA1
```
