# Supplementary material for: Expression of 5 S rRNA genes linked to 35 S rDNA in plants, their epigenetic modification and regulatory element divergence
Source: BMC Plant Biol. 2012 Jun 20;12:95. doi: 10.1186/1471-2229-12-95 (PMC3409069; doi:10.1186/1471-2229-12-95)

Page 1: clones from Artemisia, Helianthus, Tagetes and Elachanthemum

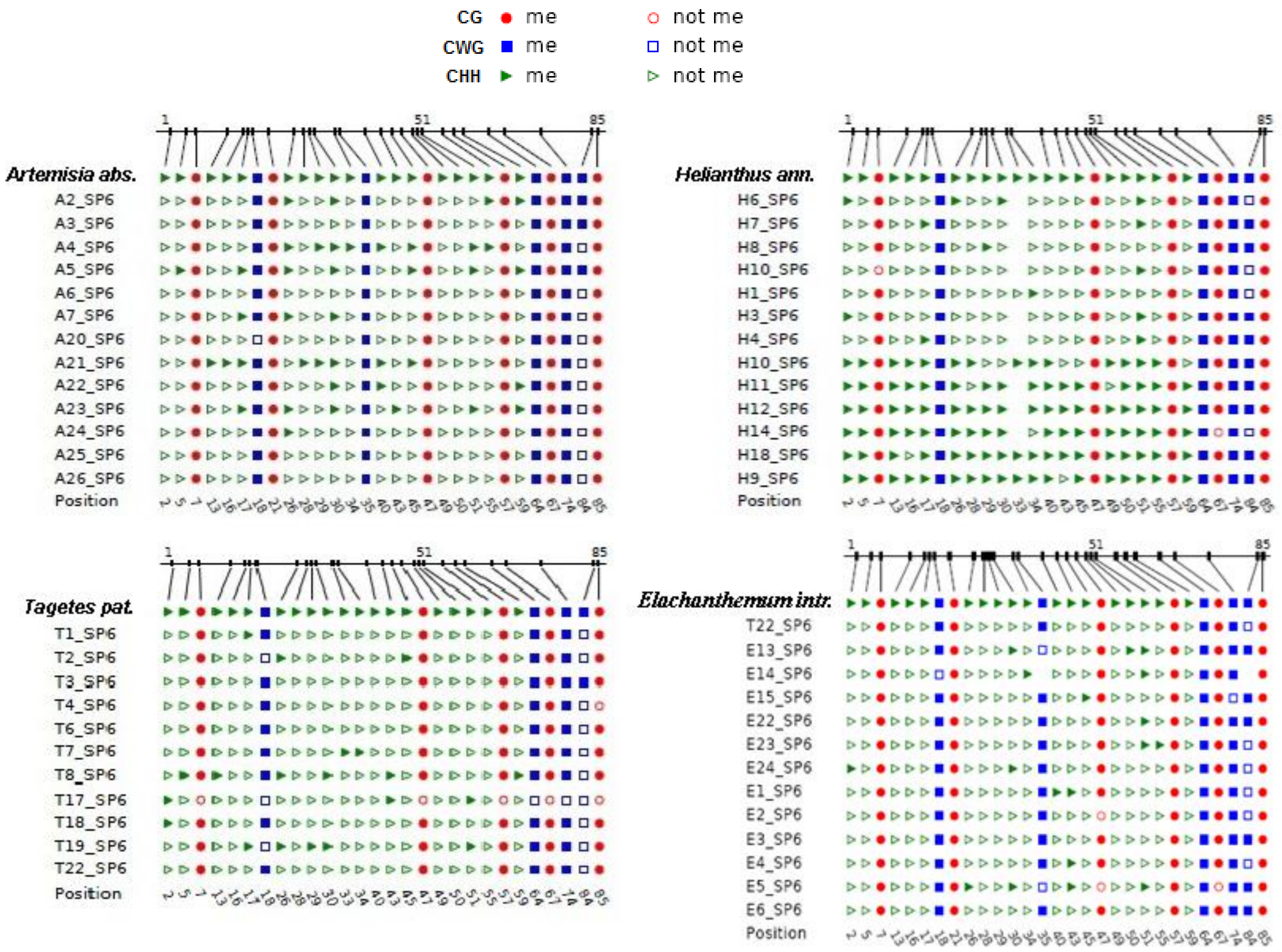

Page 2: clones from Helichrysum bracteatum

CyMATE (c) 2007, 2008

Methylation overview of 'Helichrysum_bisulfite_Cymate.afa'

Class 1: ● me ○ not me

Class 2: ■ me □ not me

Class 3: ▶ me ▷ not me

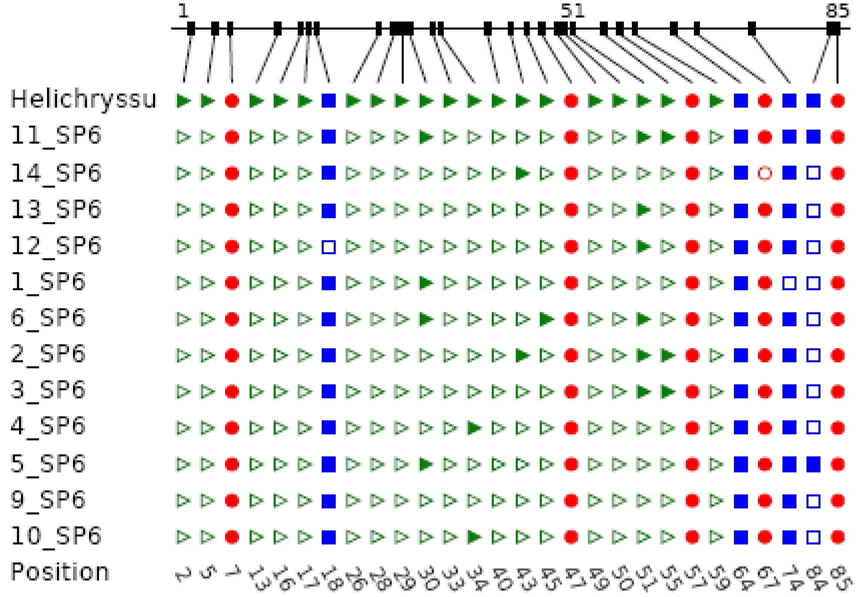

Supplement: Additional file 4 — Bisulfite analysis of the 5 S rDNA genic region(central part). Description: CyMATE program outputs from sequencing of non coding strands are shown. Filled symbols – methylated Cs; empty symbols non-methylated Cs. The numbers below the diagrams indicate C residues in the alignments. Gaps in matrices were caused by sequence polymorphisms. [file 1471-2229-12-95-S4.pdf]
